# New Frontiers for the Cytoskeletal Protein LASP1

**DOI:** 10.3389/fonc.2018.00391

**Published:** 2018-09-21

**Authors:** Elke Butt, Dayanidhi Raman

**Affiliations:** ^1^Institute for Experimental Biomedicine II, University Clinic, Wuerzburg, Germany; ^2^Department of Cancer Biology, University of Toledo Health Science Campus, Toledo, OH, United States

**Keywords:** LASP1, LASP2, transcriptional regulation, nuclear role, matrix metalloproteinases, AP1

## Abstract

In the recent two decades, LIM and SH3 protein 1 (LASP1) has been developed from a simple actin-binding structural protein to a tumor biomarker and subsequently to a complex, nuclear transcriptional regulator. Starting with a brief historical perspective, this review will mainly compare and contrast LASP1 and LASP2 from the angle of the newest data and importantly, examine their role in transcriptional regulation. We will summarize the current knowledge through pictorial models and tables including the roles of different microRNAs in the differential regulation of LASP1 levels and patient outcome rather than specify in detail all tumor entities. Finally, the novel functional roles of LASP1 in secretion of vesicles, expression of matrix metalloproteinases and transcriptional regulation as well as the activation of survival and proliferation pathways in different cancer types are described.

## Introduction

LIM and SH3 protein1 (LASP1) was originally identified in human metastatic lymph nodes from breast cancer and was named as MLN50 (1). LASP1 is located on the chromosome 17q21, a region often mutated or amplified in 20–30% of breast cancer patients and in the neighborhood of the proto-oncogene c-ERBB2 or human epidermal growth factor receptor 2 (HER2) (2) and the breast cancer susceptibility gene1 (BRCA1) (3). Though LASP1 was originally identified as a structural cytoskeletal protein, an explosive production of data in recent years provided evidence that it can orchestrate and execute multivarious roles ranging from cell signaling to transcriptional regulation.

## Comparative structural organization of LASP1 and LASP2

**Figure 1:**
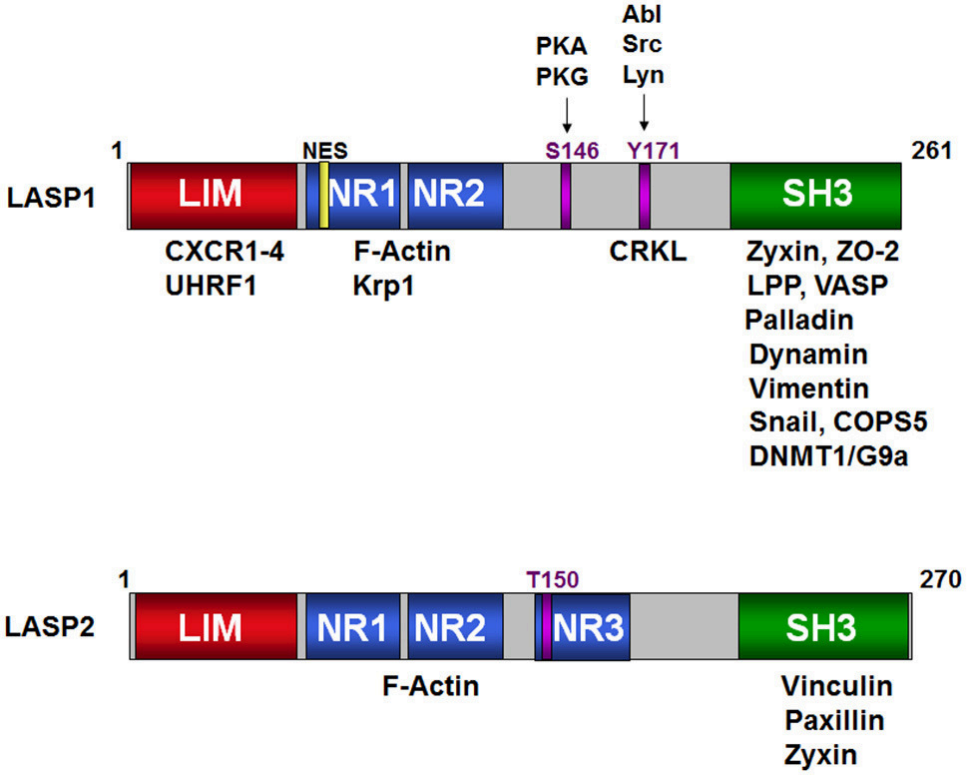
Schematic structure of LASP1 and LASP2. Binding of the different domains of LASP1 to a variety of signaling, structural, epigenetic proteins and transcription factors is shown on the top. The S146 and T171 phosphorylation sites are shown against their respective kinases. NR1, NR2, and NR3 are the nebulin repeats that bind to F-actin. Similarly, the proteins that bind to LASP2 are shown in the cartoon at the bottom. T150 is the predicted phosphorylation site in analogy to S146 in LASP1.

The mRNA for LASP1 is 4,135 bp long (NM_006148.3) and codes for a protein of 261 amino acid residues with a molecular mass of 29.7 kDa. Table 1 summarizes the similarities and the differences between LASP1 and LASP2. The LASP1 protein runs at 37–38 kDa in Western blot analysis and so far there are no structural explanations provided for this anomalous migration. Proteins with high proline content are notorious for running slower than their actual molecular mass during sodium dodecyl sulfate-polyacrylamide gel electrophoretic (SDS-PAGE) analysis. However, with a proline content of only 6.8% in LASP1, compared to 5.6% median, this does not explain the observed retarded migration in SDS-PAGE. The mRNA for LASP2 is encoded by 6,956 bp (NM_213569.2) that codes for a protein of 270 amino acid residues with a molecular mass of 31.3 kDa. LASP2 is a human ortholog of LASP1 and it was first predicted *in silico* (28) and was subsequently characterized in detail (9, 10, 14, 21). LASP1 and LASP2 are members of the large actin-binding nebulin family. However, both proteins harbor significantly lower nebulin-like repeats (NR) when compared to nebulin and nebulette and are more widely expressed (29). In addition to the characteristic NRs (two in LASP1and LASP2 and a predicted third one in LASP2) and a variable linker region, both proteins contain a *src* homology 3 (SH3) domain at the carboxyl-terminus that is absent in nebulin and nebulette (1, 8, 30, 31) (Figure 1). Phylogenetic analysis revealed an early expression of orthologous proteins in insects and invertebrates (*Bombyx mori—*silk worm, *Caenorhabditis elegans—*nematode, *Drosophila melanogaster—*fruit fly) that are ancestral to LASP1 in vertebrates (*Gallus gallus—*red jungle fowl) and mammals (*Oryctolagus cuniculus—*rabbit, *Mus musculus*—mouse, and *Homo sapiens*—Human) (32).

**Table 1:** Comparative characteristics of LASP1 and LASP2.

**Attributes**	**LASP1**	**LASP2**
Other names	MLN−50	LIM–nebulette
Size	4,135 bp—RNA 261 aa 29.67 kDa 37 kDa in SDS-PAGE	6,956—RNA 270 aa 31.2 kDa 34 kDa in SDS-PAGE
Chromosome	17q11-21.3	10p12.31
Structure	LIM-Neb-Neb-Link-SH3	LIM-Neb-Neb-Neb-Link-SH3
Expression	Ubiquitous in non-muscle tissues (4)	Brain, lung, kidney (5); Heart and skeletal muscle (6)
Localization	Focal adhesions (7); Cell membrane (8)	Focal adhesions (9) and striated muscle (Z-discs) (10)
Binding partner	F-actin (8) Zyxin (5) LPP (11); Palladin (12) Krp1(13) LASP2 (14) ZO2, (15) Dynamin (15) CXCR2/4 (16) CRKL (17) Vimentin (18) Snail1 (19) UHRF1 (19) COPS5 (20)	Zyxin (5) F-actin (21) α-actinin (10) Vinculin (10, 14) Paxillin (14) LASP1 (14)
Function	Binds and bundles actin filaments (9); Enhances cancer cell migration and cell invasion (4); Vesicular secretion (22–24)	Binds and bundles actin filaments (25); Enhances cancer cell migration but reduces cell invasion (14); Cell spreading (14, 26)
Pathology	Increased in tumors (Table 2) Nuclear localization (18, 27)	
Phosphorylation	Ser146 by PKA and PKG Tyr171 by c-Abl and c-Src	Predicted site T150—PKG over PKA

## Expression

LASP1 is ubiquitously expressed in normal human tissues (except smooth muscle) at low levels. High expression is observed in the hematopoietic system (blood cells) and the cells of the gastrointestinal tract. Interestingly, enhanced levels of LASP1 are also detected in fetal tissue, such as umbilical vein endothelial cells, fetal brain and liver, suggesting a prominent role in fetal development (4). However, LASP1 knockout mice develop normally (22) assuming a compensation for LASP1 deletion.

LASP1 is mainly located at focal adhesions (8, 7), in podosomes (33), at the leading edges of lamellipodia (9) and tips of filopodia *in vitro* and in animal models. The “Fluorescence Recovery After Photobleaching (FRAP)” approach demonstrated convincingly that the fluorescence from enhanced green fluorescent protein (EGFP)-LASP1 recovered from the base of actin bundles against the retrograde flow of actin filaments to the tip complex of the cell (9). In a similar experiment, GFP-LASP1, GFP-LASP2, and GFP-nebulette were shown to colocalize with α-actinin and vinculin at sarcomeric Z-lines or Z-disc in the periphery of spreading cardiomyocytes. However, the interaction with the A-band was only observed for LASP1 (10). LASP1 binding to F-actin and accumulation at Z-edges was also seen in *Drosophila* (10, 34), supporting the importance of this protein in evolutionary development. In contrast, localization of LASP2 at focal adhesion increased the rate of attachment and spreading for cells (26).

In several cancer types, an overexpression of LASP1 has been reported. Table 2 summarizes upregulation of LASP1 in different tumor entities. In this respect, an increased nuclear translocation of LASP1 into the nucleus inversely correlated with patient survival (i.e., poor prognosis) in breast cancer (27), prostate cancer (55), medulloblastoma (59), and hepatocellular carcinoma (18, 48). This will be discussed in detail later.

**Table 2:** LASP1 expression in human carcinoma and its regulation by microRNAs.

**Tumor entity**	**Remarks**	**References**
Breast carcinoma	LASP1 overexpression Nuclear LASP1 correlates with reduced OS miR-7 miR-203 *miR-133a*	(27, 35) (36) (37) (38)
Colorectal carcinoma	Increased LASP1 promotes metastasis; miR-133a miR-1 miR-145	(39) (40) (41) (42)
Ovarian carcinoma	LASP1 overexpression Upregulation of LASP1 in ovarian cancer	(43) (44)
Bladder cancer (BC)	Higher LASP1 expression in BC Increased urinary LASP1 in TCC patients miR-1, miR-133a and miR-218	(45) (46)
Hepatocellular carcinoma	Increased LASP1 Nuclear LASP1 expression—poor OS miR-133b	(47) (48) (49)
Esophageal squamous cell carcinoma	LASP1 overexpression—tumorigenesis miR-203 miR-203 miR-1	(50) (51) (52) (53)
Renal cell carcinoma	Overexpression of LASP1 reveals poor prognosis	(54)
Prostate carcinoma (PC)	Overexpression of LASP1 in high-risk PC miR-203 miR-1 miR-218	(55) (56) (57) (58)
Medulloblastoma	Nuclear LASP1correlates with reduced OS miR-206	(59) (60)
Nasopharyngeal carcinoma (NC)	miR-203represses LASP1 in NC	(61)
Non-small lung cancer	Overexpression of LASP1 Positive LASP1 expression correlates with worse OS miR-29 miR-203	(62) (63) (64) (65)
Gastric carcinoma (GC)	miR-219 represses LASP1 in GC cells	(66)
Lung Adenocarcinoma	Increased LASP1—reduced OS	(67)
Choriocarcinoma	Increased LASP1 expression	(68)
Gall bladder carcinoma	LASP1 overexpression	(69)
Thyroid carcinoma	High overexpression in tissue and cell lines	(70)
Pancreatic carcinoma	Overexpression is associated with poor OS	(71)

*LASP1, LIM and SH3 protein 1; TCC, Transitional Cell Carcinoma; OS, Overall survival*.

## Structure of LASP1 domains and its interacting proteins

The N-terminal cysteine rich LIM domain (residues 5–57) is composed of two zinc finger domains and functions as an adaptor for multimeric protein complexes (Figure 1, Schematic structure). LIM motif defines one class of zinc binding domains originally observed in lin-11, Isl-1, and mec-3 proteins (**L**in/**I**sl/**M**ec). Generally, LIM domain with double finger binds to two zinc ions based on spectroscopic observations (72). Solution structure of the LIM1 domain of cysteine- and glycine-rich protein 2 (CRP2) from Quail revealed that a flexible hydrophobic core of the LIM1 domain provides an optimal binding interface for its physiological targets (73). For this domain, a direct binding to the carboxyl-termini of CXC chemokine receptors 1–4 has been shown (16). While binding of LASP1 to CXCR1-3 is independent of the phosphorylation status of LASP1, the interaction with CXCR4 requires LASP1 phosphorylation at S146. Recently, binding of UHRF1 to LASP1, predominantly to the LIM domain and also in association with DNMT1 and G9a at the SH3 domain, was shown (19) (Table 3). This protein complex regulates chromatin structure and gene expression at late G1 phase and continues during G2 and M phases of the cell cycle, thus nicely explaining the observed G2/M block in LASP1 depleted cells (27, 50, 69).

**Table 3:** LASP1 binding partners and function.

**Binding partner**	**Function**	**References**
F-actin	Stabilization of F-actin bundles during cytoskeleton modulation	(8, 9)
Zyxin	Organization of the actin cytoskeleton Localization of LASP1 to focal adhesions	(5)
LPP	Organization of the actin cytoskeleton	(11)
Palladin	Localization of LASP1 to stress fibers	(12)
Krp1	Localization of LASP1 to tips of pseudopodia; necessary for pseudopodial extension and invasion	(13) (74)
CXCR1-4	Necessary for optimal ligand-mediated chemotaxis through CXC chemokine receptor	(75)
LASP2	Probably blocks the function of LASP1 through heterodimerization	(14)
ZO2	Binding to ZO-2 allows pS146-LASP1 shuttling into the nucleus	(15)
Dynamin	LASP1-dynamin interaction is reported to regulate vesicle budding and secretion	(15, 22, 23)
CRKL	Binding of pY171-LASP1 to non-phosphorylated CRKL is involved in ABL signaling	(17)
Vimentin	Co-localized with LASP1 in filopodia; involved in cell motility	(18)
Snail1	Interaction stabilizes Snail1 and mesenchymal protein expression	(19)
UHRF1-DNMT1-G9a complex	Nuclear LASP1 functions as a hub for the epigenetic machinery	(19)
COPS5	Binding to COPS5 stimulates 14-3-3 ubiquitination and degradation	(20)

*F-actin, Filamentous actin; LPP, Lipoma Preferred Partner; Krp1, Kelch related protein1; CXCR, CXC chemokine receptor; LASP1, LIM and SH3 protein 1; LASP2, LIM and SH3 protein 2; ZO2, Zona Occludens 2; CRKL, Crk-like; UHRF1, Ubiquitin-like with PHD And Ring Finger Domains 1; DNMT1, DNA methyltransferase 1; G9a, Histone H3 methyltransferase; COPS5, COP9 signalosome subunit 5; ZO2, Zona Occludens 2*.

Two nebulin-like repeats (NR) (residues 62–92 and 98–128) (R1 and R2 in Figure 1) are present in LASP1 following the N-terminal LIM domain. They vary in length (30–35 amino acid residues) and display a conserved motif: SDXXYK (76, 77). The NRs of LASP1 directly interact with filamentous actin (F-actin) (8). Photobleaching experiments with GFP-actin suggested that LASP1 is involved in actin bundling but not in polymerization of actin (9). Interestingly, in human macrophages, LASP1 has been shown to associate with annular F-actin in podosomes and to facilitate proteolysis of extracellular matrix components (33). NR of LASP1 also associates with Kelch-related protein 1 (Krp1) (13, 74) (Table 3). It remains to be elucidated if the association of Krp1 and binding of F-actin to NR of LASP1 is mutually exclusive as the authors employed whole cell lysate for the pulldown experiments and did not control for indirect Krp1 association to tagged LASP1 via F-actin binding.

**Figure 2:**
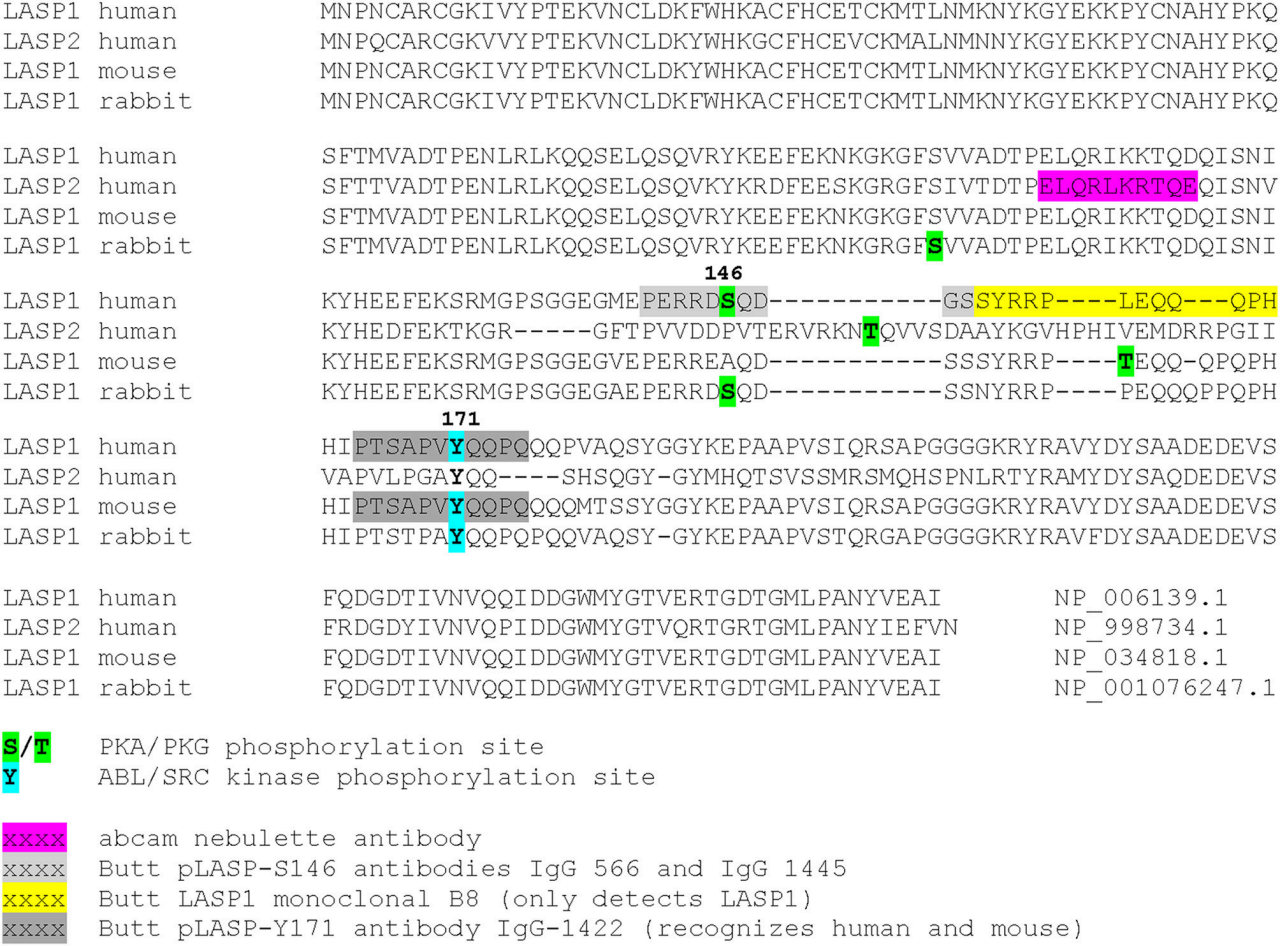
The sequence alignment of human LASP1 with its orthologous proteins. The protein sequences for human LASP1, LASP2, and mouse and rabbit LASP1 were aligned using clustalW program. The phosphorylation sites and different antibody recognition sites are color coded and depicted.

A linker region (residues 129–202) that follows NR, harbors two phosphorylation sites: S146, which is phosphorylated by protein kinase A (PKA) and protein kinase G (PKG) (78) and is dephosphorylated by protein phosphatase 2B (PP2B) (15), and Y171 that is phosphorylated by *c-Src* (79) and *c*-*Abl* non-receptor tyrosine kinases (27, 80). Phosphorylation at S146 reduces affinity of LASP1 for F-actin, zyxin, and lipoma protein partner (LPP) and allows subcellular relocalization of LASP1 to the cytosol (15, 78). In activated platelets, phosphorylation at Y171 by *c-Src* kinase leads to relocalization from focal contacts into the leading lamellae of the migrating/spreading cell. In apoptotic cells, activation of *c-Abl* prevented localization of LASP1 to focal contacts and that possibly disrupted survival signals emanating from these structures (80). Recently, phosphorylation of LASP1 at Y171 by the oncogenic BCR-ABL tyrosine kinase in chronic myeloid leukemia (CML) patients was reported. This study further demonstrated a physiological interaction between pY171-LASP1 and the src homology 2 (SH2) domain of CRK-like protein (CRKL) at amino acid sequence 36–41 that was absent under pathophysiological hyperactivation of BCR-ABL (17). In rabbit, a second PKA site at S99 was described (81); however, the sequence with just one arginine at −2 position of the phosphoserine is not an optimal consensus sequence for PKA and PKG (82). In mouse, T156 compensates for human S146 (11). The tyrosine sequence is conserved (Figure 2, Alignment). For LASP2, no functional phosphorylation sites have been reported, so far. Nevertheless, a putative consensus sequence for PKG over PKA, that resembles S146 in LASP1, is present at T150 (RKN**T**Q) in the LASP2 sequence while Y184 in LASP2, corresponding to Y171 in human LASP1, is unspecific to any tyrosine kinase when analyzed by NetPhos 3.1 program.

The carboxy-terminal SH3 domain (residues 203–261) of LASP1 binds to proteins with proline-rich motifs. Based on biochemical interaction studies, a differential comparison of proteins associating with LASP1 is shown in Table 3 and reviewed in more detail by Orth et al. (4). A recent bioinformatics approach listed putative LASP1 binding proteins mainly identified by Affinity-Capture-MS and Two-hybrid approach (83).

## LASP2 domain structure and binding partners

Like LASP1, binding of LASP2 to F-actin (9) has been demonstrated and the nebulin repeats are required for this interaction (Figure 1; Table 4). Binding of LASP2 to zyxin via SH3 domain was demonstrated by Li et al. showing an interaction between the SH3 domain of LASP1 and the PXXP-motif at the N-terminus of zyxin, localizing LASP1 to the focal adhesions (5). Recently, binding to vinculin and paxillin at focal contacts was described (14) (Table 4) and mapped to the SH3 domain of LASP2. Interestingly, the authors detected no interaction between LASP1 and vinculin or paxillin, although the SH3 domains of LASP1 and LASP2 are highly homologous (Figure 2). Furthermore, co-immunoprecipitation confirmed a binding between LASP2 and LASP1 (heterodimerization) concomitant with a reduced binding of LASP2 to vinculin and paxillin. A displacement of both proteins by LASP1 was discussed. Like LASP1, overexpression of LASP2 enhanced cell migration and cell spreading when ectopically expressed in fibroblasts (26). However, in contrast to LASP1, LASP2 is associated with reduced cell invasion (14). In this context, it is interesting to note, that high LASP1 expression was accompanied with downregulated levels of LASP2 in colorectal cancer (CRC) cell lines and tissues (84). This is in agreement with a report demonstrating enhanced expression and release of matrix metalloproteinases (MMP) by LASP1 in breast cancer cells with high metastatic potential (24). It should be noted that the rabbit polyclonal antibody against LASP2 (ELQRLKRTQE) used by Wang et al. (84) showed partial homology with the LASP1 sequence (ELQRIKKTQD). Therefore, cross-reactivity cannot be excluded and might explain in part the observed inconsistency at the translational level for LASP1 in this paper.

**Table 4:** LASP2 binding partners and function.

**Binding partner**	**Function**	**References**
Zyxin	Organization of the actin cytoskeleton Localization of LASP1 to focal adhesions	(5) (10)
F-actin	Stabilization of F-actin bundles during cytoskeleton modulation	(9)
α-actinin	Localizes LASP2 to Z-lines or Z-disc at the periphery of spreading cardiomyocytes	(10)
Vinculin	Localization of LASP2 to focal contacts Enhancing the interaction of vinculin with paxillin	(14)
Paxillin	Localization of LASP2 to focal contacts Enhancing the interaction of paxillin with vinculin	(14)
LASP1	Binding to LASP1 disrupts interaction of LASP2 to vinculin and paxillin	(14)

*LASP1, LIM and SH3 protein 1; LASP2, LIM and SH3 protein 2; F-actin, Filamentous actin; Z-lines, Lateral boundaries of the basal contractile unit of the cardiomyocyte*.

A similar problem occurred in the recent paper by Zhang et al. when studying the effect of LASP2 on non-small cell lung cancer (NSCLC) and phosphorylation of focal adhesion kinase (FAK) (85). The used antibody, claimed to be specific for LASP2 was actually generated against nebulette (NEBL). The immunogen sequence given by the company does not correspond in any part to LASP2. Therefore, the data should be handled with precaution. Overall, there are clear functional differences between LASP1 and LASP2 (see Table 1) which also explain the fact that LASP2 did not compensate for LASP1 knockout in mice (22).

## Regulation of LASP1 expression

There is strong evidence that LASP1 is upregulated in tumors under hypoxic conditions (86, 87). Hypoxia response elements were identified in the LASP1 promoter and were shown to stimulate LASP1 expression in pancreatic cells *in vitro* and in mouse tumor xenografts (71). Furthermore, several reports demonstrate an overexpression of LASP1 in response to microRNA (miRNA) downregulation (Table 2). Prediction of microRNA target sequences at the 3‘UTR region of LASP1 by “*TargetScanHuman ver 7.1”* revealed more than 15 putative miRNAs including miR-145, miR-218, miR-133a, miR-1, miR-29, miR-218, and mainly miR-203 that have been connected to LASP1 overexpression (Table 2). Conversely, in preeclampsia, hypoxia induces miRNA-218 expression, resulting in a downregulation of LASP1 and inhibition of trophoblast invasion (87).

Besides the regulation by HIF1-α and miRNAs, LASP1 is also regulated by the tumor suppressor p53, at least in hepatocellular carcinoma (88), gastric cancer (89), and endometrial cancer (90). As 50% of human tumors show somatic mutations in p53, loss of p53 activity might account for LASP1 overexpression in several cancer types, however, not all tumors with a defect in p53 show increased LASP1 levels (27). A bioinformatics approach identified Foxa1 and Foxa2 binding sites in the LASP1 promoter region, however, whether these two transcription factors are capable of regulating LASP1 expression remains to be elucidated (18). Recently, the presence of a consensus-binding site for SOX9 in the promoter region of LASP1 was detected. Luciferase reporter and ChIP assays verified LASP1 transcriptional regulation by SOX9 (91).

## Functional roles of LASP1 in cancer

### Vesicular secretion

Since the discovery of LASP1 in 1995, the protein was observed to be overexpressed in a variety of tumor types (Table 2) and the list is still increasing. Interestingly in a melanoma study, LASP1 was described to be distinctly expressed only in the basal epidermal layer of the normal skin and in melanocytes while in primary melanoma and in metastases, a reduced LASP1 expression was noticed and no nuclear presence could be detected (23). The immunofluorescence approach identified LASP1 to be co-localized with dynamin and visualized an overlapping with melanosomes at the tips of melanocyte dendrites. It is assumed, that LASP1 is part of the F-actin-dynamin mediated budding of melanosome-containing vesicle into the extracellular matrix (92). Knockdown of LASP1 led to a distinct reduction in melanosome vesicle shedding in normal human epidermal melanocytes (NHEM) cells.

**Figure 3:**
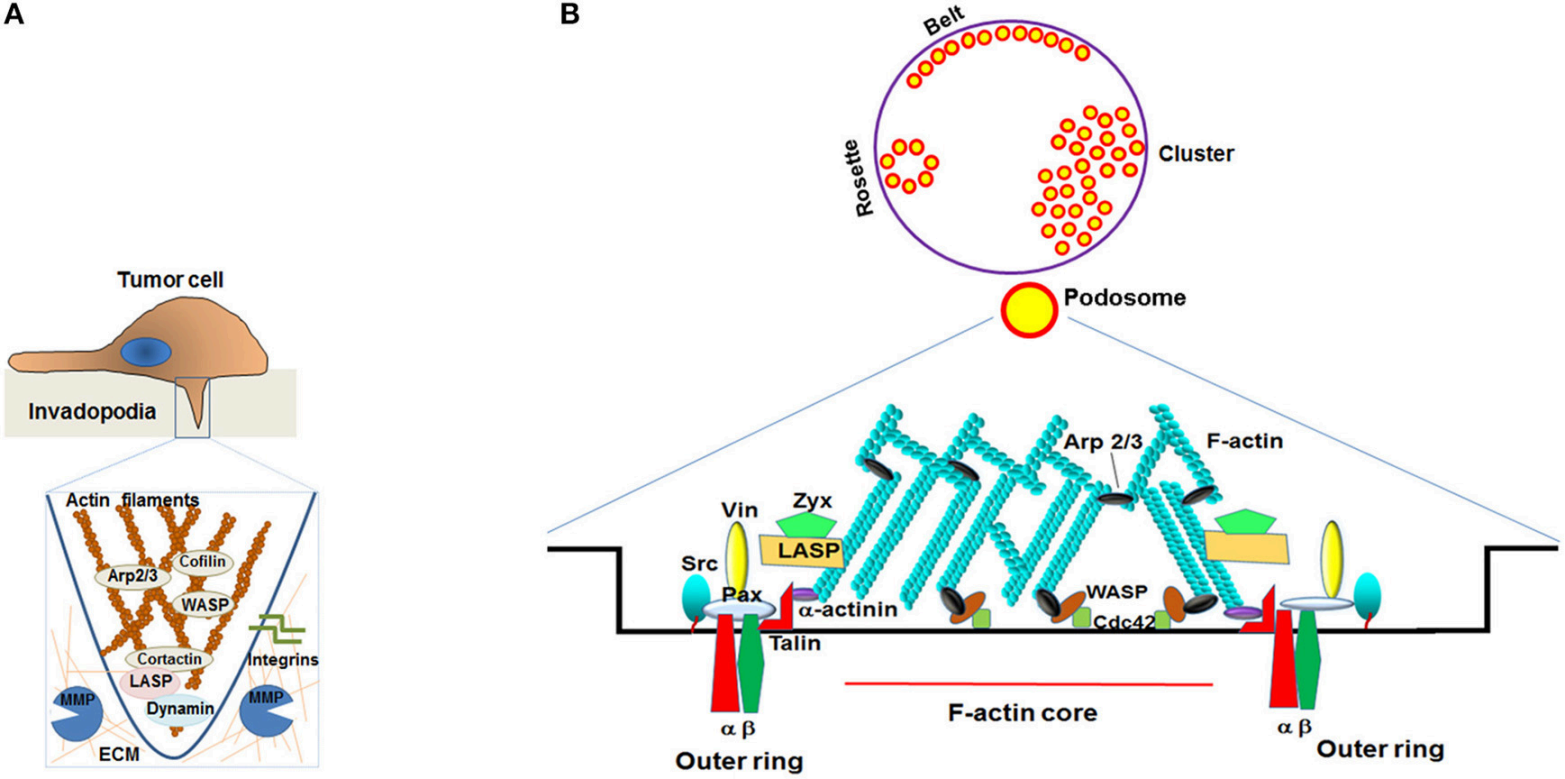
Schematic representation of MMP secretion from invadopodia and podosomes. **(A)** Schematic representation of the invadopodia: During tumor invasion, invadopodia promote the degradation of the extracellular cell matrix (ECM) by coordinating the secretion of matrix metalloproteases MMP1, MMP3 and MMP9 at the tip of the protruding structure. The detailed mechanism is still unknown but involvement of LASP1 and dynamin is discussed. **(B)** Schematic representation of the podosome: The top picture depicts the different patterns of distribution of the podosomes (belt, rosettes, and clusters). An expanded view of a single podosome is shown below. LASP1was documented to co-localize with zyxin and vinculin in human macrophages in the outer ring area but not at the inner F-actin core. Vin, Vinculin; Zyx, Zyxin; Pax, Paxillin; LASP, LASP1 or LASP2; WASP, Wiskott-Aldrich syndrome protein; Arp 2/3, Actin-related protein 2/3.

A similar mechanism is assumed for LASP1 in vesicular secretion of matrix metalloproteinases (MMPs) (24). Again, a complex of LASP1 with dynamin and F-actin, is observed and studies promote a fractionated release of vesicular MMPs into the surrounding tissue, endowing cancer cells with increased invasiveness (Figure 3A). Depletion of LASP1 led to reduced MMP levels that are rescued or restored after LASP1 overexpression (24).

Earlier, LASP1 was observed to have an impact on hydrochloric acid (HCl) secretion in gastric parietal cells. The authors suggested a phosphorylation-dependent alteration of LASP1 binding to F-actin, ezrin, and dynamin as likely mediators linking the vesicular trafficking/activation of H^+^,K^+^,-ATPase to the cytoskeleton (22). It remains to be seen whether this would play a role in gastric cancer.

### LASP1 and matrix metalloproteinases

Matrix metalloproteinase (MMPs) are proteolytic enzymes capable of degrading extracellular matrices (ECM) like collagen, elastin, and fibronectin and therefore are involved in cell proliferation, migration, differentiation, angiogenesis, apoptosis, and defense. During tumor cell metastasis, cells disrupt cadherin-based intercellular junctions and initiate detachment from the primary site. This is enhanced by MMPs which digest the basal lamina components and facilitates cell movement through the ECM (93). In this respect, LASP1 plays a pivotal role in tumor invasion and metastasis by releasing MMPs into the ECM via specialized cell membrane domains called invadopodia (Figure 3A) *akin to* podosomes in normal cells (94). Stolting et al. showed that LASP1 colocalizes with zyxin and vinculin in the podosome ring structure of human macrophages though LASP1 has not been shown to bind to vinculin biochemically (Figure 3B). Subsequently, knockdown of LASP1 affected podosome dynamics and impaired matrix degradation capacity in these cells (33). Recent studies provided evidence that stable silencing of LASP1 *also* reduced gene expression levels of MMP9 and 1 in MDA-Bone-Un breast cancer cells (MDA-MB-231 cells that were re-isolated from mouse bone metastatic lesions) (19). This was further supported by reports indicating that transient knockdown of LASP1 led to reduced MMP1, 3 and 9 expression in MDA-MB-231 breast cancer cells by affecting the MMP transcription factor AP1 (24). Reduced MMP1 levels after LASP1 depletion were also observed in LNCaP prostate cancer and T24 bladder cancer cell lines (24) suggesting a general role of LASP1 in favoring distant metastasis by enhanced transcription and secretion of MMPs from invadopodia (Figure 3A).

### Nuclear function

**Figure 4:**
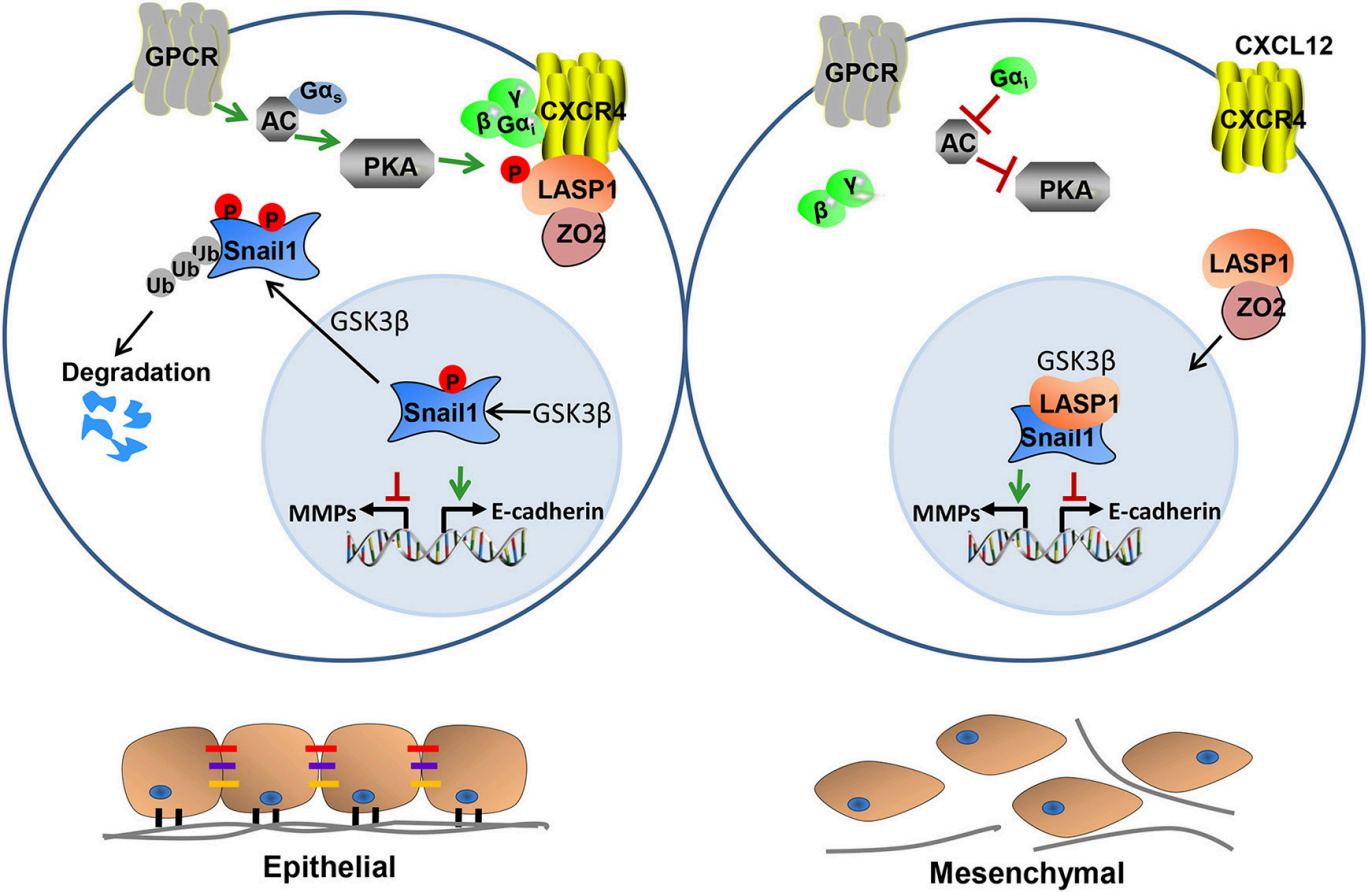
Proposed model for LASP1-Snail1 interaction. A model has been depicted in which a G-protein coupled receptor (GPCR) coupled to Gα_s_ activates protein kinase A (PKA) that in turn phosphorylates LASP1 on S146 and facilitates its binding to CXCR4. Upon activation of CXCR4 by its ligand CXCL12, Gα_i_ is activated and inhibits adenylyl cyclase (AC)/PKA signaling thus preventing accumulation of pS146-LASP1. Upon dephosphorylation of CXCR4, bound phospho-LASP1 is released and co-imported with ZO2 into the nucleus. There, LASP1 stabilizes Snail1 to impart its epigenetic silencing of E-cadherin expression leading to mesenchymal, migratory, and invasive morphology which is typical of CXCR4 activation.

In 2007, Grunewald et al. (35) first reported a nuclear localization of LASP1 in 29% of breast carcinoma patients and later on nuclear localization was correlated with a reduced overall survival rate of invasive breast tumor patients (27). Nuclear LASP1 localization was confirmed in several breast cancer cell lines where the protein increased at S-phase and peaked at a G2/M phase (27). Likewise, a small proportion of medulloblastoma samples showed a nuclear localization of LASP1 (59). Recently, nuclear LASP1 distribution was also described for hepatocellular carcinoma (HCC) (48) and is observed in human bladder cancer tissue (46). As LASP1 only harbors a nuclear export signal (NES) within amino acid sequence 71–77 (Figure 1), the protein is reliant on a nuclear shuttle partner. Phosphorylation of LASP1 at S146 reduces the binding to F-actin and zyxin at the membrane and induces translocation of LASP1 from the cytoplasm into the nucleus through the interaction with nuclear shuttling protein zona occludens 2 (ZO2) (15). There, it was shown to bind to Snail1 and a stabilization of the protein by LASP1 was discussed (19). As Snail forms a transcriptional repressor complex and lowers gene expression of adherens junction proteins like E-cadherin and occludin, stabilization of this complex may enhance mesenchymal cell formation and induce tumor cell migration (95) (Figure 4). Furthermore, the work by Duvall-Noelle et al. showed association of LASP1 with UHRF1 and G9a in a CXCL12-dependent manner, assuming that LASP1, in addition, serves as a nuclear hub for the epigenetic machinery in breast cancer cells (19). Recently, this work was confirmed by data showing an enhanced Snail expression and decreased E-cadherin levels also in NSCLC after LASP1 overexpression (63).

There is growing evidence that LASP1 can also act as a transcriptional co-factor. Microarray analysis of MDAMB-231 breast cancer cells before and after LASP1 depletion revealed 39 regulated genes, thereof 8 (22%) regulated by AP-1 (24). AP-1 is a heterodimer that comprises members of the proto-oncogene c-Jun and c-Fos protein family and may form ternary complexes with transcriptional co-factors (96). AP-1 luciferase reporter assay confirmed LASP1-inducible AP-1 response, however, the authors failed to show a direct binding of LASP1 to either c-Jun or c-Fos or regulation of c-Fos protein levels assuming a more complex regulation.

### LASP1, S100A, 14-3-3, PI3K/AKT, and COPS5—some critical remarks

During the last years, the group by Liang Zhao from the Nanfang Hospital in Guangzhou, China, published several papers suggesting a fundamental role for LASP1 in epithelial-mesenchymal transition (EMT) by enhancing or modulating the expression of several proteins involved in EMT, like S100A4 (97), S100A11 (98), 14-3-3σ (99), and COPS5 (20). These studies suggest an interaction between LASP1 and aforementioned proteins, however, for evidence mainly co-localization of LASP1 with S100A11, 14-3-3σ, and COPS5 in colorectal cancer (CRC) cells is shown. The reported associations of LASP1 described for S100A11 or COPS5 lack proof for a direct interaction with LASP1. Direct binding of COP5 was only shown to the truncated SH3 domain of LASP1 but not to the full-length LASP1 protein and the proposed (but not shown) LASP1/14-3-3σ protein complex would require LASP1 phosphorylation as 14-3-3σ binding is primarily serine phosphorylation dependent (100) Most likely, the observed interactions are indirect by association to a ubiquitous protein like F-actin that is often pulled down using Sepharose beads in these experiments, with high interference observed especially with the paramagnetic beads and F-actin or glutathione S-transferase (GST)-tags. The same holds true for the proposed LASP1-FAK interaction shown in NSCLC (63). Here, a weak but detectable binding of FAK to IgG control is observed and therefore, a direct relationship between LASP1 and FAK has to be confirmed.

**Figure 5:**
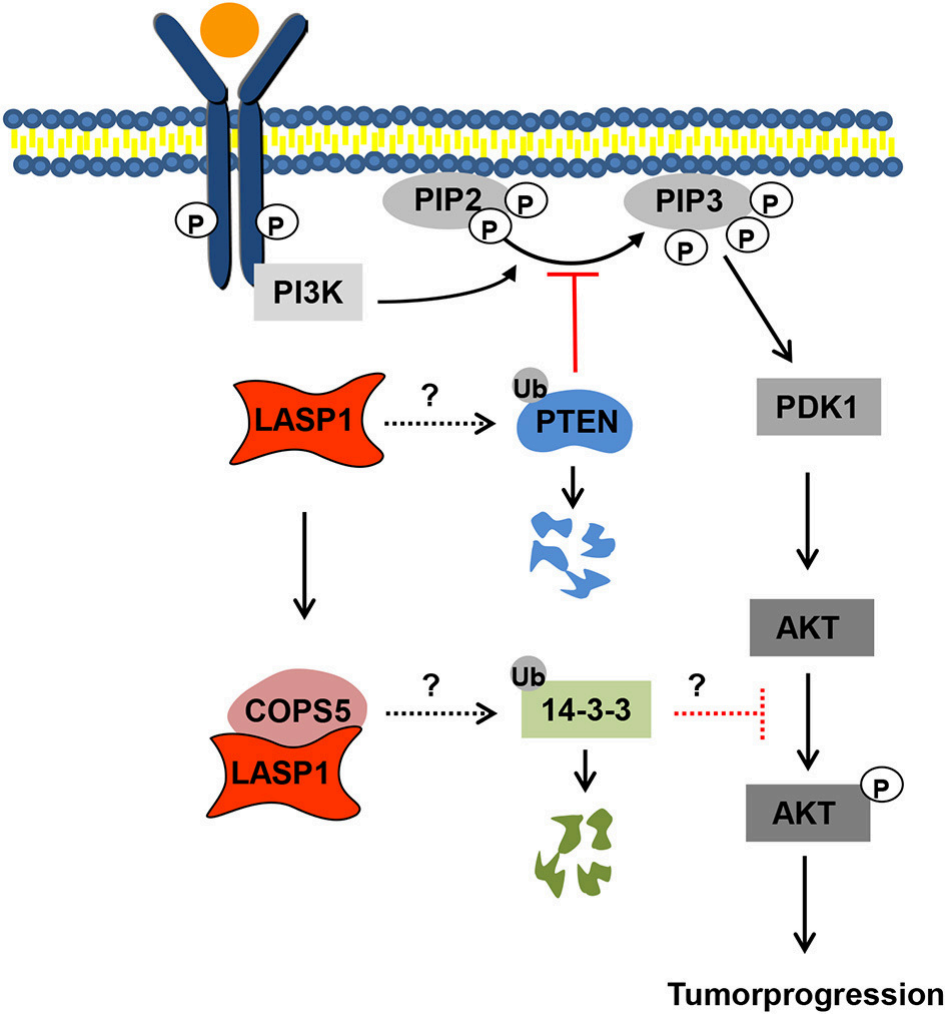
Proposed model for LASP1-PTEN-COPS5-14-3-3σ interaction. Model illustrate discussed LASP1/AKT signaling pathways. 14-3-3σ somehow inhibits AKT activation. LASP1 and COPS5 (COP9 signalosome subunit 5) synergistically stimulate ubiquitination and degradation of 14-3-3σ, resulting in AKT-S473 phosphorylation. In a second hypothesis, LASP1 enhances ubiquitination and degradation of PTEN (phosphatase and tensin homolog), thus enhancing PIP2 phosphorylation to PIP3 and concomitant AKT activation.

Concerning AKT, several papers discussed an influence of LASP1 on PI3K/AKT pathway. Depletion of LASP1 resulted in decreased pAKT-S473 phosphorylation while overexpression of LASP1 showed enhanced pAKT accumulation (20, 69, 99, 101). It has been suggested that an inverse correlation of the expression levels between LASP1-COPS5 complex and 14-3-3σ somehow affects the phosphorylation of AKT at S473. However, the exact molecular mechanisms underlying the activation of AKT by LASP1 are unclear. In Figure 5, the LASP1 promoted effects on PI3K/AKT activation are schematically presented (99). Very recently, an association of LASP1 with the phosphatase and tensin homolog (PTEN) was shown by co-immunoprecipitation but co-localization in nasopharyngeal carcinoma cells was not convincing due to big nuclei and a thin rim of cytoplasm (102). The authors claim an inverse correlation of the protein levels between LASP1 and PTEN by immunohistochemistry and that again suggested a LASP1-dependent degradation of PTEN through ubiquitination which may allow an enhanced activation of ligand-activated PI3K pathway (Figure 5) (102).

## Author contributions

EB constructed all tables and figures except Figure 3B which is drawn by DR.

### Conflict of interest statement

The authors declare that the research was conducted in the absence of any commercial or financial relationships that could be construed as a potential conflict of interest.
